# A Pedigree-Based Map of Recombination in the Domestic Dog Genome

**DOI:** 10.1534/g3.116.034678

**Published:** 2016-09-02

**Authors:** Christopher L. Campbell, Claude Bhérer, Bernice E. Morrow, Adam R. Boyko, Adam Auton

**Affiliations:** * Department of Genetics, Albert Einstein College of Medicine, Bronx, New York 10461; † New York Genome Center, New York 10013; ‡ Department of Biomedical Sciences, College of Veterinary Medicine, Cornell University, Ithaca, New York 14853

**Keywords:** canine genetics, crossover interference, hotspots, PRDM9, recombination

## Abstract

Meiotic recombination in mammals has been shown to largely cluster into hotspots, which are targeted by the chromatin modifier PRDM9. The canid family, including wolves and dogs, has undergone a series of disrupting mutations in this gene, rendering *PRDM9* inactive. Given the importance of *PRDM9*, it is of great interest to learn how its absence in the dog genome affects patterns of recombination placement. We have used genotypes from domestic dog pedigrees to generate sex-specific genetic maps of recombination in this species. On a broad scale, we find that placement of recombination events in dogs is consistent with that in mice and apes, in that the majority of recombination occurs toward the telomeres in males, while female crossing over is more frequent and evenly spread along chromosomes. It has been previously suggested that dog recombination is more uniform in distribution than that of humans; however, we found that recombination in dogs is less uniform than in humans. We examined the distribution of recombination within the genome, and found that recombination is elevated immediately upstream of the transcription start site and around CpG islands, in agreement with previous studies, but that this effect is stronger in male dogs. We also found evidence for positive crossover interference influencing the spacing between recombination events in dogs, as has been observed in other species including humans and mice. Overall our data suggests that dogs have similar broad scale properties of recombination to humans, while fine scale recombination is similar to other species lacking *PRDM9*.

The placement of recombination events within the genome is not random, but instead is concentrated into regions known as hotspots. Recent work in humans and mice has identified the protein PRDM9 as responsible for targeting recombination events to hotspots (Baudat *et al.* 2010; Myers *et al.* 2010; Parvanov *et al.* 2010). PRDM9 is active early in meiotic prophase (Hayashi *et al.* 2005) and contains a zinc finger (ZF) array that binds to specific DNA motifs located at hotspot centers. Upon DNA binding, PRDM9 trimethylates lysine 4 on histone H3, and is presumed to recruit cellular machinery to initiate recombination through an unknown mechanism.

Recombination hotspots are a common feature in many eukaryote genomes for which data are available, but are not typically conserved between species. For example, humans and chimpanzees have a complete absence of hotspot sharing, despite a high degree of overall DNA sequence identity (Ptak *et al.* 2005; Winckler *et al.* 2005; Auton *et al.* 2012). This change in hotspot location appears to be driven by the rapid evolution of the ZF domain of *PRDM9*, which is subject to strong selection in primates and rodents as well as a variety of ancient metazoans (Oliver *et al.* 2009). Alterations to the ZF domain modify DNA motif recognition and binding specificity (Oliver *et al.* 2009) and hence contribute to a shifting landscape of active hotspots in the genome.

Evidence from mice suggests that PRDM9 is required for the proper completion of meiosis. Loss of *PRDM9* causes sterility in male mice due to impairment of the progression of early meiotic prophase (Hayashi *et al.* 2005). These mice, despite being sterile, still initiate double-strand breaks (DSBs), and these breaks cluster into hotspots. However, there is almost no overlap with hotspots that occur in mice with functional PRDM9, and these DSBs occur preferentially at promoters and CpG-rich regions of the genome (Brick *et al.* 2012). This pattern is similar to that in other species lacking *PRDM9*, including birds (Singhal *et al.* 2015), and yeast (Lam and Keeney 2015).

The canid ortholog of *PRDM9* has been inferred to have undergone multiple truncating mutations in the last exon, encoding the ZF array, and become a nonfunctional pseudogene (Muñoz-Fuentes *et al.* 2011). These mutations are shared within the Canidae family that includes dogs, coyotes, wolves, and foxes and must have accumulated after their divergence from pandas, who do not share the mutations, approximately 49 MYA (Oliver *et al.* 2009; Muñoz-Fuentes *et al.* 2011; Axelsson *et al.* 2012; Auton *et al.* 2013). Nonetheless, these species are all able to complete meiosis and reproduce, which implies either that the function of PRDM9 in dogs is replaced by another gene or that recombination is completed successfully in its absence. A rare homozygous loss of function mutation in *PRDM9* has been recently reported in humans, in which a healthy mother was found to have mutations predicted to abolish both methyltransferase and DNA binding activity (Narasimhan *et al.* 2016), leading to reduced crossover activity at PRDM9-dependent hotspots. This mother had three healthy children, suggesting that humans may be able to successfully complete meiosis and remain fertile without functional PRDM9.

Despite the lack of *PRDM9*, hotspot-like regions of recombination have been inferred in dogs from patterns of linkage disequilibrium (LD). These hotspots differ qualitatively from those found in humans, appearing to have a lower intensity of recombination rate, and covering a wider genomic interval (∼4–18 kb compared with ∼2 kb in humans) (Axelsson *et al.* 2012; Auton *et al.* 2013). However, direct comparisons are complicated by differences in the general LD properties of the species arising from, for example, population demography (Auton *et al.* 2013). Most striking is the observation that recombination is preferentially targeted toward CpG-rich regions, such as those found in gene promoter regions, which is similar to recombination patterns found in other species without *PRDM9*.

The rate of recombination is known to differ between the sexes in many species, a phenomenon known as heterochiasmy (Mank 2009). Other features of recombination are known to differ between males and females, and collectively contribute to a general sexual dimorphism in recombination. In humans, for instance, males are known to have a higher rate of recombination at the telomeres (Coop *et al.* 2008), and similar observations have been made in the dog genome (Wong *et al.* 2010). Human recombination additionally appears elevated just outside of gene regions, but depressed within them (Kong *et al.* 2010). Some of these effects have been observed in dogs, but fine scale studies of sex-specific recombination have been limited. The intriguing finding of promoter-concentrated recombination in dogs is limited to LD studies (Auton *et al.* 2013), raising questions as to whether a direct ascertainment of recombination could uncover further details.

In addition, data on features of recombination that cannot be assessed from LD studies, such as crossover interference, is lacking in dogs. Previous linkage studies of dog recombination have relied on low-coverage genotyping, making inference of interference difficult. One earlier study used cytological methods to estimate interference in the dog genome, finding evidence for positive interference of a similar magnitude to that of other mammals (Basheva *et al.* 2008). Further characterization of this phenomenon would serve to increase our understanding of recombination in this unique species.

Over the last three decades, the study of recombination in dogs has progressed from initial low-coverage linkage maps (Mellersh *et al.* 1997; Neff *et al.* 1999), bolstered by the assembly of a draft sequence of the dog genome (Lindblad-Toh *et al.* 2005), to higher-coverage pedigree maps (Wong *et al.* 2010), and high-resolution LD based maps from single nucleotide polymorphism (SNP) array and whole genome sequence data (Axelsson *et al.* 2012; Auton *et al.* 2013). Here, we present a pedigree analysis of recombination in the domestic dog, *Canis lupus familiaris*. The genetic maps from this study represent an advance over previous linkage maps by using high-density SNP microarray data and the latest build of the dog genome. These maps provide a valuable resource to the canine genetics community, and allow investigation of the sex-specific distribution of recombination in the dog genome. Given the open questions regarding the role of PRDM9 in recombination, we compared the sex-specific landscape of recombination in the dog genome to that inferred from human pedigrees, and used this comparison to gain further insight into the effects of the presence or absence of PRDM9 on the mammalian recombination landscape.

## Materials and Methods

### Genotyping

The full dataset is derived from genomic analysis of 237 DNA samples, with 25 founder individuals (15 male, 10 female, pedigree structure shown in Supplemental Material, Figure S1) from a colony of Labrador Retriever and Greyhound crosses maintained at Cornell University for over 30 yr (Todhunter *et al.* 2003; Mateescu *et al.* 2008; Phavaphutanon *et al.* 2009). Genotyping was performed using genomic DNA as described in Hayward *et al.* (2016) using Illumina CanineHD BeadChips that include more than 170,000 SNPs. All positions reported are given in canFam3.1 coordinates.

### Filtering SNP data

To avoid spurious recombination calls due to genotyping error, we applied a set of filters on the variant data (outlined in Figure S2). Starting with the set of 166,171 autosomal SNPs, we first remove 586 SNPs because they had missing genotypes in > 5% of the samples. We then used the PLINK (Purcell *et al.* 2007) software (v1.07) to identify and remove a further 1245 SNPs showing Mendelian errors in transmission (option --mendel). The error detection feature in the Merlin (Abecasis *et al.* 2002) software (v1.1.2, option --error) was used to identify and remove SNPs with genotypes that conflicted with pedigree structure and are likely to be genotyping errors. Three iterations of Merlin error detection were performed, removing 1363, 66, and 7 SNPs, respectively. In total, 2771 unique SNPs were filtered out in the first round, leaving 163,400 SNPs for further analysis.

### Calling crossover events

Autosomal recombination events were inferred using a combination of software tools. First, the dog genomes were phased without using pedigree information using SHAPEIT2 (Delaneau *et al.* 2013) (v2.r790). In order to avoid bias in our inference of recombination, we use a map file for phasing that has a constant rate of recombination (1 cM/Mb) between physical markers. Following phasing, we used the duoHMM (O’Connell *et al.* 2014) software (v0.1.4) to call recombination events using a hidden Markov model approach. The duoHMM software uses information on the relatedness between individuals to improve the quality of the phased haplotypes from SHAPEIT2. From these haplotypes, the inheritance pattern is inferred and recombination events are identified. The first duoHMM pass integrated pedigree structure information to correct phasing errors. Then, duoHMM was used to call crossovers in each parent–child duo of the pedigree, of which only high-confidence events were retained (with probability > 0.5). The duoHMM method was also used to identify SNPs that have a high probability of genotyping error (which we removed if a SNP had a probability of error of > 0.9). This method has been demonstrated to have a high sensitivity and low false discovery rate when compared to a standard Lander–Green (Lander and Green 1987) approach, such as that implemented in Merlin (Abecasis *et al.* 2002).

### Filtering crossovers

Tightly clustered crossovers within individuals may occur naturally as a result of gene conversion; however, another possibility is that genotype errors have caused false crossover calls. In many cases in our data, we found double crossovers that belonged to a shared parent and are clustered within the genome. Furthermore, these crossovers often used the same SNP for the interval boundaries. This strongly suggests genotyping error as a likely cause. Therefore, we removed any double crossovers that cluster within 1 Mb, and if more than one meiosis transmitted from the same parent also had clustered crossovers with shared interval boundaries.

We further removed all crossovers attributed to meioses that had biologically abnormal crossover counts (Figure S3). We defined thresholds separately for males and females, with a distribution centered on the median crossover count, and defined the boundaries as ± 4 SD from the sex-specific median crossover count, with the SD estimated via the robust estimator of 1.4826 median absolute deviation (MAD).

### Construction of the genetic map

Due to the high level of inbreeding and homozygosity in domestic dogs, it is often not possible to detect recombination events over a significant fraction of the genome within a given pedigree. For example, if a breeding pair has few heterozygous variants toward the telomeric ends of a given chromosome, then events occurring within such regions will be largely invisible as they will not be flanked by informative markers. Due to the high level of inbreeding within many dog samples, failure to account for this issue would result in an underestimate of the total map length. To correct for this issue, we considered the location of informative markers within each pedigree, and scaled the genetic map accordingly.

In order for duoHMM to correctly identify a recombination event, it must be flanked by at least one heterozygous variant in the parent on each side. For each parent–child duo for which we were able to make crossover calls, we identified the positions of the first and last heterozygous variant on each chromosome, which represented the genomic range in which we were able to observe a crossover. Then, across all duos in our sample, we estimated the effective total number of meioses at each position along each chromosome (Figure S4). The effective number of meioses was used in place of a fixed number of meioses when calculating the recombination fraction at each genomic interval.

Recombination fractions were converted to genetic distances using Haldane’s map function (Haldane 1919). Comparing maps generated using the effective number of meioses to those using a fixed number of meioses, we observed an increase in autosomal map length for both females (59.7 cM) and males (49.2 cM), and an increase in the sex-averaged map length of 48.6 cM (Figure S5).

### Estimation of crossover interference parameters

Crossover interference influences the spacing of crossover events when two or more occur on the same chromosome in the same meiosis. We modeled the distance between these crossovers using two models. The gamma model (Broman and Weber 2000) assumes that the intercrossover distances follow a simple gamma distribution with shape *ν* and rate 2ν, where *ν* is a unitless measure of the strength of crossover interference, with ν = 1 representing no crossover interference, ν < 1 representing negative interference (spaced closer than expected by chance), and ν > 1 indicating positive crossover interference (spaced further apart than expected). The gamma-escape model, originally proposed by Housworth and Stahl (2003) provides an extension to the simple gamma model. Here, crossovers that are governed by the interference effect (ν > 1) are modeled to coexist alongside a subset of crossovers that escape interference (ν = 1). A second parameter, *p*, is included to allow the second class of “escaping” crossovers to exist in a mixture with the interfering crossovers, and represents the proportion of events that escape interference. To measure crossover interference, we estimated the parameters *ν* and *p* using a MATLAB software package (https://github.com/auton1/interference) previously developed to analyze interference in humans (Campbell *et al.* 2015).

In order to compare each of the fitted models, we used the Bayesian Information Criterion (BIC), which is given by: BIC = −2ln(*L*) +*k*ln(*n*), where *L* is the maximum likelihood estimation from the model fit, *k* is the number of free parameters in the model, and *n* is the number of observations. The model with the smallest BIC is preferred.

### Gene annotations

Gene annotations for canFam3.1 were downloaded from Ensembl (build 81). We considered only protein coding genes located on the autosomes, and kept the longest isoform for each gene.

### Thinning the human map

In order to make a valid comparison between dogs and humans with respect to the proportion of recombination occupying a given amount of sequence, we took a number of steps to ensure the datasets are as similar as possible. To enable sex-specific comparisons between the species, we used a human pedigree dataset (Campbell *et al.* 2015) rather than an LD-based map. As the human dataset is considerably larger, we randomly sampled 408 phase-known meioses (204 each from males and females) to match the size of the dog dataset. We then reduced the SNP density of the human data. The human dataset was genotyped on a microarray of higher density than we have available for dogs, which allows recombination events to be better resolved than what was possible for dogs. Therefore, we thinned the human dataset in an attempt to match the recombination event resolution in dogs using an *ad hoc* iterative process as follows: (1) determine the inter-SNP distances for the human and dog datasets, (2) find SNPs that cluster more tightly in humans, (3) remove a random subset of SNPs within each of these clusters, and (4) iterate until the inter-SNP distributions and overall medians are similar between the two species. This iterative process yields a thinned framework of SNPs in the human dataset such that the new inter-SNP distances closely resemble those in dogs (Figure S6). Following this thinning, we examined each individual crossover in the human data and expanded the interval boundaries, if necessary, to the next available SNP in the newly selected framework. New sex-specific genetic maps were then generated from this thinned data and used for the comparison to dogs.

### Estimating the concentration of recombination within the genome

We used the genetic maps to estimate the how concentrated recombination is across the genome. Having removed large physical gaps (such as those around the centromeres), physical intervals were sorted by the recombination rate in descending order. We then plotted the proportion of recombination as a proportion of physical distance. C.I. for these estimates were obtained through a bootstrap approach in which chromosomes were sampled with replacement 1000 times.

### Data availability

Supplementary data for this study, including sex-specific genetic maps, filtered crossover calls, and genotype data are available at https://github.com/clcampbell/dog_recombination. 

## Results

### Building the genetic map

We used SNP array genotype data from 237 domestic dogs to map recombination in the canine genome. After applying a series of filtering steps on the SNP data (Figure S2), we identified crossovers using duoHMM, a tool previously developed to identify recombination events in human data (O’Connell *et al.* 2014). Upon examination of the initial genetic maps, we identified several regions that exhibited biologically unrealistic recombination rates within concentrated physical regions and could be errors. Several of these regions overlap known segmental duplications and copy number variants (Nicholas *et al.* 2009; Chen *et al.* 2009), which could account for the observed clustering of crossovers. Another possible explanation is that the reference genome contains misplaced or inverted contigs within these regions, which would lead to calling of false crossover events on either side of the out-of-place region. We removed all 435 variants within four such regions, totaling 5.6 Mb of sequence (Table S1), and a further 1344 markers identified with the error detection feature of duoHMM.

After recalling crossovers on this filtered data, we found 8312 autosomal recombination events. We then identified and removed a subset of clustered double crossovers, likely to be false calls, consisting of 90 events. Finally, we excluded meioses that have a biologically abnormal number of crossovers, excluding three female meioses (with 55, 41, and 119 crossovers), and three male meioses (with 5, 44, and 109 crossovers). In total, we excluded 463 out of 8312 crossovers (5.6%).

The filtered dataset consisted of 161,699 autosomal markers, with an average of 44,067 (95% C.I.: 42,262–45,872) informative markers per meiosis. There were 408 informative meioses, including 204 from females and 204 from males. There are 7849 well-supported crossover events that could be localized to a median size of 102.1 kb (Figure S7). The sex-specific genetic maps had a mean resolution of 0.35 cM (female) and 0.36 cM (male).

### Comparison to previous studies

To assess the accuracy and validity of our results, we compared our maps to those from previous studies. We used sex-specific maps from a previous pedigree analysis (Wong *et al.* 2010), as well as a sex-averaged LD-based map generated from whole genome sequencing data of 51 village dogs (Auton *et al.* 2013). At the broad scale, there is close agreement to our sex-averaged map from the LD map (Pearson *r* = 0.86 at 5 Mb resolution), and from the pedigree sex-averaged map (*r* = 0.75, Figure S8A). The male map has a higher agreement with previous studies (LD *r* = 0.80, pedigree *r* = 0.76, Figure S8B) than does the female map (LD *r* = 0.67, pedigree *r* = 0.735, Figure S8C).

Consistent with previous studies in dogs (Wong *et al.* 2010; Mellersh *et al.* 1997; Neff *et al.* 1999) and other mammals, including humans (Coop *et al.* 2008; Kong *et al.* 2010; Campbell *et al.* 2015), females have a longer map length (2162 cM) than males (1816 cM, Table 1 and Table S2). We observed similar total genetic map lengths when compared to the Wong *et al.* (2010) pedigree study, although our maps are slightly shorter (by 114 cM in females, 93 cM in males, Table 1). Map length is strongly correlated to physical length in both sexes (male *r*^2^ = 0.82, female *r*^2^ = 0.83), and in the sex-averaged maps (*r*^2^ = 0.88; Figure S9). The ratio of female to male autosomal map length is 1.19, equivalent to Wong *et al.* (2010), but notably lower than that of humans at 1.6 (Campbell *et al.* 2015).

**Table 1:** Autosomal map length estimates

Study	Year	Female (cM)	Male (cM)	Ratio	Sex Avg. (cM)
Mellersh *et al.* (1997)	1997	1039	766	1.36	902.5
Neff *et al.* (1999)	1999	1820	1290	1.41	1555
Wong *et al.* (2010)	2010	2276	1909	1.19	2092.5
Axelsson *et al.* (2012)	2012				3005
Auton *et al.* (2013)	2013				2430
This study	2016	2162	1816	1.19	1978

Total map lengths are given in centimorgans, while the ratio represents the female-to-male map lengths. Sex-specific map lengths are not available for the LD-based maps. Avg., average; LD, linkage disequilibrium.

### Distribution of recombination

**Figure 1:**
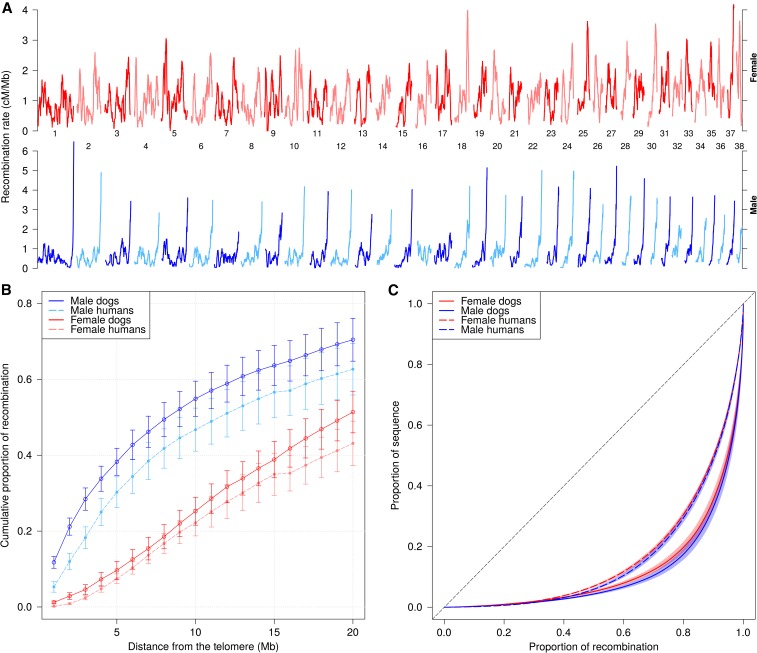
The distribution of recombination across the genome. (A) Broad scale recombination rates differ between males and females. Rates were smoothed at the 5 Mb scale. Chromosomes 27 and 32 are likely reversed in the canFam3.1 genome build, and are shown here with their physical coordinates reversed. (B) The proportion of recombination as a function of the distance from the telomeric end of each chromosome arm. Error bars represent a 95% C.I. (C) Proportion of recombination occupying various fractions of the sequence. The human data were thinned to match the SNPdensity and meiosis count of the dog dataset. The colored bands for each curve represent 95% C.I. calculated from 1000 bootstrap iterations. For all panels, males and females are shown in shades of blue and red, respectively. Human data in panels (B) and (C) is shown in dashed lines.

Previous studies showed that the recombination rate is elevated in telomeric regions and lower near the centromere, both in dogs (Wong *et al.* 2010; Axelsson *et al.* 2012; Auton *et al.* 2013) and other species (de Massy 2013). We observed the same phenomenon, and this telomeric effect is largely driven by male recombination in both dogs and humans (Figure 1A and Figure S10). The opposite pattern is seen in two chromosomes, 27 and 32, supporting previous evidence (Wong *et al.* 2010) suggesting that the orientation of these chromosomes in the reference genome is likely reversed. Based on this, we reverse the physical coordinates for these two chromosomes for all further analyses.

We quantified the amount of recombination occurring at the telomeric ends of each chromosome arm by estimating the proportion of total recombination occurring in a physical window located at the telomeric end (Figure 1B). We found that 38.2% of recombination occurred within 5 Mb of the telomere in males, compared with 9.7% in females. Human males have a roughly equivalent proportion within this same region (30.2% within 5 Mb), while human females have a similar amount of recombination compared to female dogs (7.4% within 5 Mb). We conclude that, at a broad scale at least, the telomeric enrichment of recombination observed in male dogs is similar to that of humans.

Previous observations using LD recombination maps have raised the possibility that dog recombination may be more uniform in distribution throughout the genome than in humans due to the loss of *PRDM9* and its associated hotspots (Axelsson *et al.* 2012; Auton *et al.* 2013). However, estimates from LD can be confounded by differences in the effective population size, which complicate such comparisons. Pedigree-based studies should not be subject to the same confounding issues, and to investigate this further, we examined the concentration of recombination rates across the genome using our pedigree maps and compared this to human pedigree data (Campbell *et al.* 2015). This analysis is sensitive to the marker coverage and crossover resolution, so the human genetic maps have been reduced in resolution to match that of the dog data (see *Materials and Methods*, Figure S11, A and C). We found that 80% of recombination occurred in a smaller proportion of the dog genome [17.5% (95% C.I.: 15.5–19.5) male, 19.8% (95% C.I.: 18.2–21.7) female, Figure 1C] than previously reported in LD-based estimates. In contrast to the LD-based findings (Figure S12), dog recombination is actually less uniform than in the human thinned data, in which males have 29.5% (95% C.I.: 28.0–31.0), and females 30.7% (95% C.I.: 30.0–31.6) of their sequence containing the majority (80%) of recombination. In addition, males of both species have slightly more focused recombination when compared to females, although there is overlap in the C.I. To address the possibility that differences in genome architecture or recombination rate distribution could account for these observations, we excluded telomeric regions from the analysis, and matched dog chromosomes with similarly sized human chromosome arms (Figure S11, B and D), with similar results. Therefore, it appears that crossovers are more concentrated within a smaller proportion of the dog genome than in humans, and that this effect is more pronounced in males of both species.

### Recombination around genomic features

Starting from the observation that recombination is targeted to CpG islands concentrated at gene promoter regions (Auton *et al.* 2013), we looked for these effects in our data, and to what extent they are sex-specific. Using the genetic maps, we estimated recombination rates in nonoverlapping 10 kb bins across the genome. We found that recombination rates were elevated around the TSS, both in the sex-averaged and male maps, but no peak was discernible in females (Figure S13A and Table S3). Additionally, the male recombination rate in the surrounding regions was higher than in females, despite a lower genome wide recombination rate. This observation can be partially explained by a modest enrichment in the number of genes (31%) occurring in the telomeric 25% of each chromosome, where male recombination is more frequent. However, while the male rate is higher overall in telomeric regions, males exhibited a peak at the TSS even in nontelomeric regions (Figure S14, A and B).

Both male and female recombination estimates showed elevated recombination surrounding CpG islands (Figure S13B and Table S3). The peak in male dogs was higher than females by 0.98 cM/Mb, with a high rate in the surrounding sequence, which could be explained by clustering of CpGs, as well as an enrichment of CpG islands in telomeric, male driven recombination regions (42 in 25% of sequence, Figure S14, C and D). To control for the effects of CpG clustering and concentration at the telomeres, we analyzed a thinned set of CpG islands, obtained by randomly selecting a maximum of five CpG islands per nonoverlapping 500 kb window. Using this thinned, uniformly distributed set of CpG islands, we found that the male and female background rates were more comparable. However, the male peak remained higher, suggesting that recombination around CpG islands is dominated by males (Figure S15). We also examined recombination rates around H3K4 trimethylation marks found via ChIPseq on dog spermatocytes (Auton *et al.* 2013). As previously reported, the presence of these marks associated with elevated recombination rates; however, this association can be explained by the proximity of CpG islands to H3K4me3 marks (Figure S16), and we saw no differences between males and females.

### Crossover interference

Crossover interference, a phenomenon that affects the physical spacing between pairs of crossover events occurring during the same meiosis, acts in various species, including humans (Campbell *et al.* 2015; Broman and Weber 2000; Housworth and Stahl 2003), mice (Broman *et al.* 2002), and cattle (Sandor *et al.* 2012). To learn more about interference in dogs, we examined the distribution of intercrossover distances in our dataset. We fitted two models of crossover interference, the gamma model (Broman and Weber 2000) and the gamma-escape model (Housworth and Stahl 2003) (also known as the Housworth–Stahl model). The gamma-escape model is a mixture model that builds upon the gamma model, adding a subset of events that escape interference.

**Figure 2:**
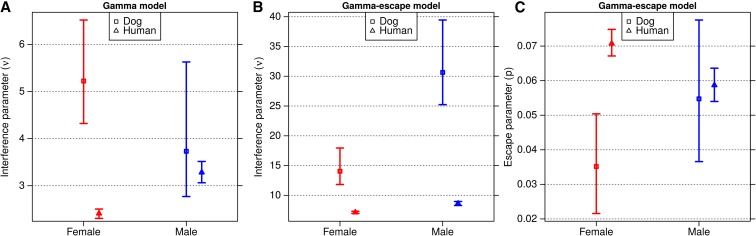
Estimates of crossover interference parameters in the dog genome using the simple gamma model (A) and Housworth–Stahl gamma-escape model (B and C). Panels (A) and (B) show the interference strength parameter, *ν*, for each model, while the right panel (C) shows the escape parameter, *p*, the proportion of events that escape interference. Males are shown in blue and females in red, while estimates for dogs are shown in boxes, and humans in triangles. The error bars represent a 95% C.I. estimated from 1000 bootstrap iterations.

The no-interference model (ν = 1), had a poor fit, with a lack of double crossovers in close proximity, indicating that positive crossover interference must be acting to some degree in dogs. When fitting the simple gamma model, estimates of interference strength in male and female dogs overlapped with each other. These estimates are comparable to a cytological study in dogs measuring the distance between MLH1 foci, which mark crossovers (*ν* = 6.5) (Basheva *et al.* 2008). Compared to humans, female dogs have a stronger strength of interference than human females, while the estimates for males of both species overlap (Figure 2A and Table 2).

**Table 2:** Autosomal crossover interference

	Gamma Model		Gamma-Escape Model		
	*ν* (95% C.I.)	BIC	*ν* (95% C.I.)	*p* (95% C.I.)	BIC
Male dogs	3.73 (2.77–5.63)	6617.1	14.05 (11.82–17.95)	0.055 (0.037–0.078)	6174.6
Female dogs	5.22 (4.32–6.52)	7580.9	30.64 (25.23–39.44)	0.035 (0.022–0.050)	7274.0
Male humans	3.28 (3.06–3.51)	99,043.7	8.63 (8.29–8.96)	0.059 (0.054–0.064)	93,205.3
Female humans	2.41 (2.31–2.50)	168,775.7	7.13 (6.95–7.33)	0.071 (0.067–0.075)	156,793.4

Parameter estimates are shown for both gamma and gamma-escape models for combined autosomes in dogs and humans. Numbers in parentheses represent 95% C.I. BIC, Bayesian Information Criterion.

In the interference-escape model, estimates of the strength of interference across the dog genome are higher in males than in females (ν_female_ = 14.05, ν_male_ = 30.64). This trend is similar to that seen in humans, with stronger crossover interference in males; however, the parameter estimates are higher by a factor of two in females, and more than three in males (ν_female_ = 7.19, ν_male_ = 8.93 in humans, Figure 2B). In contrast, male dogs have a similar proportion of escaping events to humans (5.5 *vs.* 5.9%), while female dogs have fewer events (3.5%) escaping than human females (7.1%, Figure 2C). We found support for both models of interference in the dog dataset; however, we used BIC to make a formal comparison of the goodness of fit for each model. We found that in both sexes, the gamma-escape model is preferred over the simple gamma model (Table 2), in agreement with previous findings supporting a two-pathway model of crossover interference in humans (Housworth and Stahl 2003; Campbell *et al.* 2015).

To test if this difference reflects a change in interference parameters with parental age, as is observed in humans (Campbell *et al.* 2015), we divided our dataset by age into seven approximately equal sized bins. No differences were observed for crossover parameters for either model in any of the age groups (Figure S17). However, we should note that bootstrapped estimates produce large error bars and there is insufficient power to adequately detect any age differences in this dataset.

When reducing the resolution of the human dataset (see *Materials and Methods*), we found that the parameter estimates were largely unchanged compared to those of the full resolution data, although with wider C.I. (Figure S18). This provides confidence that parameter estimation using these models is robust to crossover interval size resolution and dataset power, and that the parameters estimated from our dog data are likely to accurately reflect those of this dataset.

## Discussion

Since the discovery of PRDM9 and its importance to recombination, questions about its full role have persisted. While *PRDM9* is under selection across a variety of species (Oliver *et al.* 2009), a notable subset are missing a functional version of this protein, raising questions regarding the landscape of recombination in these species. Our pedigree study adds to existing work and provides insight into recombination in the absence of *PRDM9*. Our findings confirm that fine scale recombination in dogs tends to cluster into hotspot-like regions near gene promoters. Conversely, broad scale patterns appear largely conserved between dogs and other studied mammals, with greater female recombination rate overall, and substantially higher rates at the telomeres in males.

On a broad scale, our results are in agreement with those from previous studies, demonstrating that dog recombination is similar to other mammals, indicating that the presence or lack of *PRDM9* does not change broad scale patterns of crossover placement. In particular, a majority of crossing over occurs in telomeric regions in males, while female crossing over is both more frequent and more spread out.

We report a ratio of female to male map lengths of 1.19, equivalent to previous estimates from Wong *et al.* (2010). This groups domestic dogs with a large collection of species that exhibit sexual dimorphism in recombination in which the female has a higher rate of crossing over, including humans (Coop *et al.* 2008; Kong *et al.* 2010) and mice (Cox *et al.* 2009). In contrast, cattle are one of the few species that have the opposite trend, with a recent study in domestic cattle estimating the male map length to be 10% longer than females (Ma *et al.* 2015). An interesting suggestion from this study was that the overall recombination rate in males may have been affected by artificial selection pressure. Because artificial selection is more frequently focused on males, this can result in an increase in recombination if selection acts positively on recombination rate. If true, it is not implausible that this selective pressure could have altered recombination in dogs during their domestication as well, something that could be revealed through a comparison to wolves, the closest ancestor to the modern domestic dog.

Initial estimates using LD maps in dogs indicated that 80% of all recombination falls into a fairly large (30–46%) amount of sequence (Axelsson *et al.* 2012; Auton *et al.* 2013), markedly more spread out than the < 20% figure seen in human LD maps (The International HapMap Consortium 2007). This supports the idea that PRDM9 acts to funnel recombination into hotspots in humans, and supports the hypothesis that dog recombination, lacking this hotspot-specifying protein, is more uniform across the genome. While further investigation is necessary, our findings here, using pedigree data, suggest that dog recombination may actually be less uniform than humans. Furthermore, in both species, males appear slightly more focused than females. This effect in humans could potentially be explained by a higher male hotspot usage (Campbell *et al.* 2015). In dogs, this could be due to higher male rates around gene promoter regions and CpG islands that are concentrated toward the telomeres. This concentration of recombination at functional genomic elements is not unique to dogs, but appears to be shared among a number of species lacking *PRDM9*, including *PRDM9* knockout mice (Brick *et al.* 2012), *Arabidopsis*, yeast (Lam and Keeney 2015), and birds (Singhal *et al.* 2015).

The concentration of recombination at these functional elements supports a working model for *PRDM9*-absent species, in which recombination occurs preferentially in regions of open chromatin. Another implication is that recombination hotspots in dogs and other *PRDM9*-absent species may be stable in evolutionary time, in contrast to current evidence against hotspot sharing in *PRDM9*-dependent species. Since dog hotspots lack a strong motif that is likely to be targeted by a *trans*-acting factor such as PRDM9 (Axelsson *et al.* 2012; Auton *et al.* 2013), they are not likely to be subject to the hotspot paradox that acts to continually erode the binding capacity of hotspots, even as they are actively being used for recombination (Myers *et al.* 2010). Evidence for hotspot stability has been found in two finch species, which share recombination hotspots that appear to be separated by tens of millions of years (Singhal *et al.* 2015), as well as four yeast species sharing hotspots over 15 million years of evolution (Lam and Keeney 2015).

The distribution of intercrossover distances in dogs supports the existence of positive crossover interference in the dog genome, with similar estimates of interference strength under the simple gamma model to those in humans. Nonetheless, our results favor the gamma-escape model, supporting the idea that two separate pathways contribute to recombination in dogs. This two-pathway model of interference appears to be conserved between humans and dogs, and suggests that broad scale recombination properties are similar between the two species regardless of PRDM9 status. Interestingly, while an increase in interference escape with age has been observed in human females (Campbell *et al.* 2015), no such age effect was observed in dogs. Accepting that our canine sample size would limit our ability to detect such effects, another potential explanation is that, in contrast to humans, the timing of meiotic events in dogs is substantially different. Recombination in human females begins and enters a potentially lengthy meiotic arrest prenatally, resuming just prior to ovulation. In contrast, meiosis in female dogs begins later, in the neonatal period. While recombination is complete prior to ovulation in humans, dogs ovulate immature oocytes, after which meiosis must complete before the oocyte becomes fertile, around 48 hr after ovulation (Freixa *et al.* 1987; Chastant-Maillard *et al.* 2011).

Overall, these results add to a growing body of research in nonhuman recombination genetics, and provide a step toward answering many open questions in canine recombination. Further work is needed on larger and more diverse pedigrees, both in domestic dogs and other members of the Canidae family, including wolves, in order to form a more complete picture of recombination in this family.

## Supplementary Material

Supplemental Material
