# Supplementary material for: A Pedigree-Based Map of Recombination in the Domestic Dog Genome
Source: G3 (Bethesda). 2016 Sep 2;6(11):3517–24. doi: 10.1534/g3.116.034678 (PMC5100850; doi:10.1534/g3.116.034678)
Supplement: Supplemental Material [file supp_6_11_3517__index.html]

A Pedigree-Based Map of Recombination in the Domestic Dog Genome — Supplemental Material 

# A Pedigree-Based Map of Recombination in the Domestic Dog Genome

## Supplemental Material for Campbell *et al.*, 2016

**Files in this Data Supplement:**

- Table S1 - Regions removed from the dataset. (.pdf, 40 KB)
- Figure S7 - Distribution of crossover interval size. (.pdf, 35 KB)
- Figure S8 - Pearson correlation between recombination rates estimated from the Auton *et al*. LD map, the pedigree maps from Wong *et al*., and this study as a function of scale. (.pdf, 61 KB)
- Figure S9 - Map length as a function of physical length for each chromosome for female (A), male (B), and sex-averaged (C) maps. (.pdf, 59 KB)
- Figure S10 - Recombination rate across the human genome using the 23andMe genetic maps. (.pdf, 293 KB)
- Figure S11 - SNP density affects the proportion of recombination occupying various proportions of the sequence. (.pdf, 1 MB)
- Figure S12 - Estimates for the proportion of recombination occupying various proportions of the sequence differ between LD- and pedigree-based maps. (.pdf, 501 KB)
- Figure S13 - Sex differences in recombination around the TSS (A) and CpG islands (B). (.pdf, 48 KB)
- Figure S14 - Recombination around TSS and CpG islands partitioned by chromosome position. (.pdf, 46 KB)
- Figure S15 - Recombination around a thinned subset of CpG islands. (.pdf, 36 KB)
- Figure S16 - Recombination rate around H3K4 trimethylation marks found in dog spermatocytes of varying stages. (.pdf, 66 KB)
- Table S2 - Physical and genetic chromosome lengths. (.pdf, 60 KB)
- Figure S17 - Estimates of crossover interference parameters in the dog genome as a function of age. (.pdf, 39 KB)
- Figure S18 - Estimates of crossover interference parameters in the human genome. (.pdf, 38 KB)
- Table S3 - Recombination rate around TSS and CpG islands. (.pdf, 39 KB)
- Figure S1 - Structure of the dog pedigree. (.pdf, 41 KB)
- Figure S2 - Overview of the analysis pipeline. (.pdf, 116 KB)
- Figure S3 - Distribution of the number of crossover events per meiosis. (.pdf, 84 KB)
- Figure S4 - The effective number of meioses as a function of physical position is shown along each chromosome. (.pdf, 49 KB)
- Figure S5 - Increase in map length in each chromosome after accounting for the effective number of meioses. (.pdf, 35 KB)
- Figure S6 - Distribution of inter-SNP distances in the dog data. (.pdf, 37 KB)
