# Supplementary material for: A Pedigree-Based Map of Recombination in the Domestic Dog Genome
Source: G3 (Bethesda). 2016 Sep 2;6(11):3517–24. doi: 10.1534/g3.116.034678 (PMC5100850; doi:10.1534/g3.116.034678)
Supplement: Supplemental Material [file supp_g3.116.034678_TableS1.pdf]

Chromosome	Start	End	Size	# variants
6	44745965	47085070	2339105	182
16	53878711	56703603	2824892	221
19	20011075	20320803	309728	24
32	38654394	38810281	155887	8
		**Total**	**5629612**	**435**

Table S1: Regions removed from the dataset.
