# Supplementary material for: A Pedigree-Based Map of Recombination in the Domestic Dog Genome
Source: G3 (Bethesda). 2016 Sep 2;6(11):3517–24. doi: 10.1534/g3.116.034678 (PMC5100850; doi:10.1534/g3.116.034678)
Supplement: Supplemental Material [file supp_g3.116.034678_FigureS8.pdf]

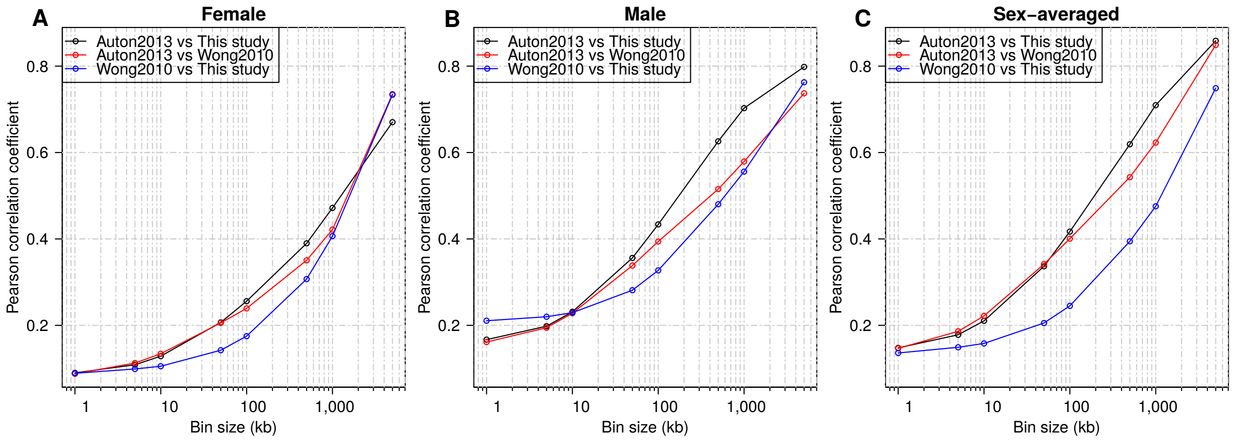


Figure S8: Pearson correlation between recombination rates estimated from the Auton *et al.*[1] LD map, the pedigree maps from Wong *et al.*[2], and this study as a function of scale.
