# Supplementary material for: A Pedigree-Based Map of Recombination in the Domestic Dog Genome
Source: G3 (Bethesda). 2016 Sep 2;6(11):3517–24. doi: 10.1534/g3.116.034678 (PMC5100850; doi:10.1534/g3.116.034678)
Supplement: Supplemental Material [file supp_g3.116.034678_FigureS9.pdf]

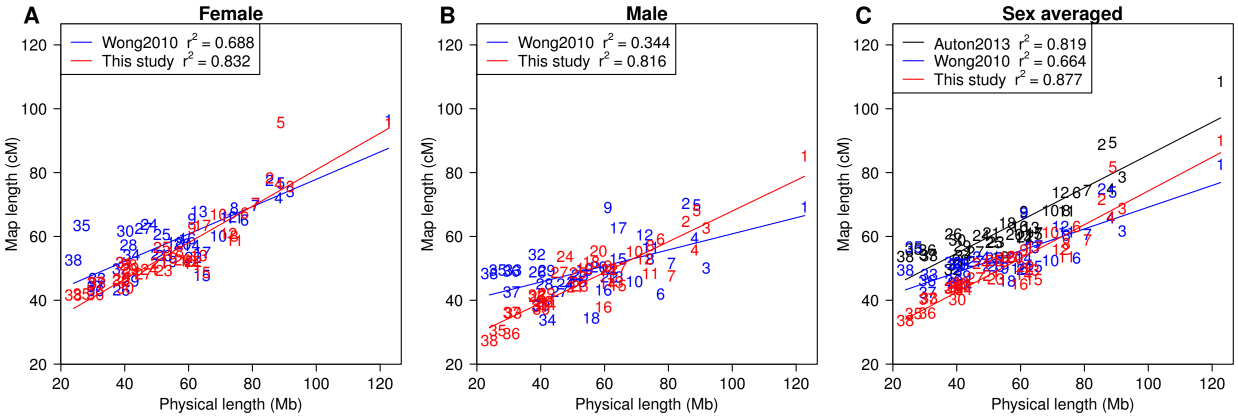

Figure S9: Map length as a function of physical length for each chromosome for female (A), male (B), and sex-averaged (C) maps. Numbers refer to chromosomes with a linear regression line included. The sex-specific maps are compared to the Wong *et al.*[2] pedigree study, the sex-averaged map is additionally compared to the LD map from Auton *et al.*[1] (C, in black).
