# Supplementary material for: A Pedigree-Based Map of Recombination in the Domestic Dog Genome
Source: G3 (Bethesda). 2016 Sep 2;6(11):3517–24. doi: 10.1534/g3.116.034678 (PMC5100850; doi:10.1534/g3.116.034678)
Supplement: Supplemental Material [file supp_g3.116.034678_FigureS10.pdf]

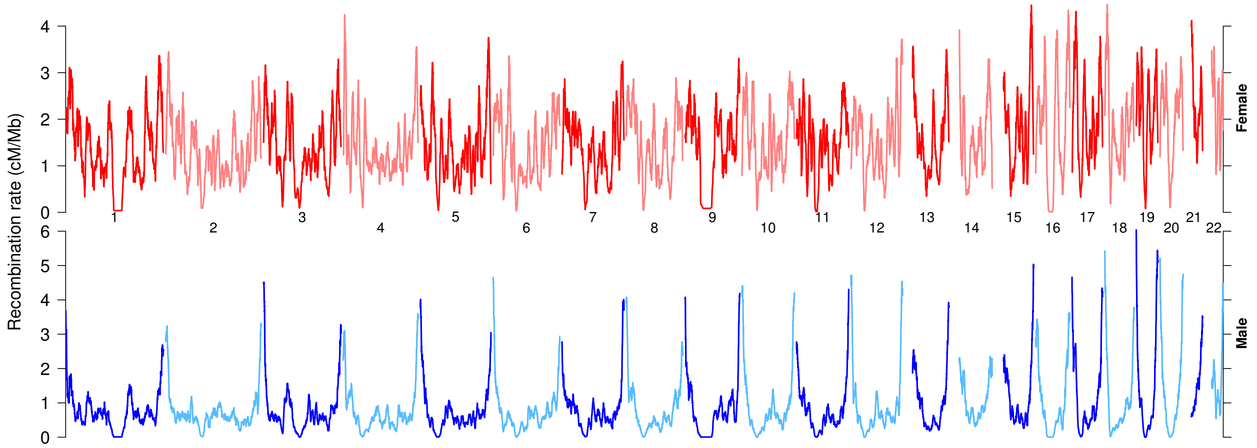

Figure S10: Recombination rate across the human genome using the 23andMe genetic maps[3]. Female rates are shown in shades of red, male rates in blue shades. Recombination rates were smoothed at the 5 Mb scale.
