# Supplementary material for: A Pedigree-Based Map of Recombination in the Domestic Dog Genome
Source: G3 (Bethesda). 2016 Sep 2;6(11):3517–24. doi: 10.1534/g3.116.034678 (PMC5100850; doi:10.1534/g3.116.034678)
Supplement: Supplemental Material [file supp_g3.116.034678_FigureS11.pdf]

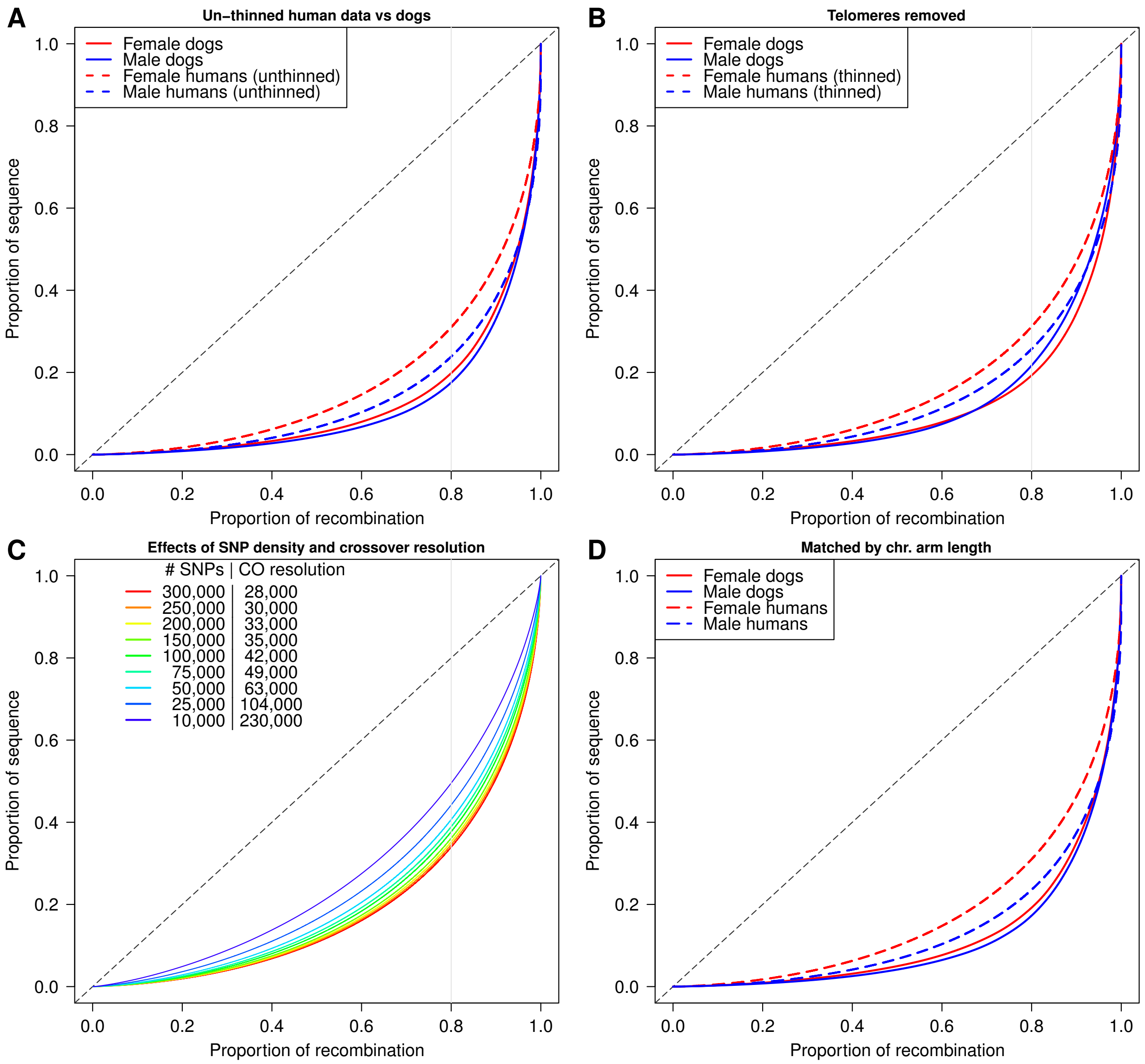

Figure S11: SNP density affects the proportion of recombination occupying various proportions of the sequence. (A) Human data is shown prior to thinning, alongside data from dogs. Even without thinning in humans, dogs have a more concentrated distribution of recombination. (B) Dog data is shown with the thinned human data. Here, the most telomeric 15% (by physical distance) of each chromosome arm has been excluded for both species. Dog recombination remains more concentrated than humans, although the female and male dog curves have flipped at the 80% recombination mark, likely as a result of the removal of large amounts of telomeric recombination in males. (C) The effects of thinning the SNP framework used to create the genetic maps for the human data. Each curve represents a different marker density, from 300,000 SNPs (red line), to 10,000 (blue line), with corresponding crossover resolutions indicated in the legend. Reducing the SNP density and crossover resolution moves the curve closer to unity and causes recombination to appear to be more spread out throughout the genome. (D) A reduced set of human (un-thinned) and dog data are shown with the chromosome sizes approximately matching. Each dog chromosome was paired with a corresponding human chromosome arm of a similar size (within 30 Mb). This plot includes dog chromosomes 1 through 28; the remainder were too small to have potential matching human chromosome arms.
