# Supplementary material for: A Pedigree-Based Map of Recombination in the Domestic Dog Genome
Source: G3 (Bethesda). 2016 Sep 2;6(11):3517–24. doi: 10.1534/g3.116.034678 (PMC5100850; doi:10.1534/g3.116.034678)
Supplement: Supplemental Material [file supp_g3.116.034678_FigureS12.pdf]

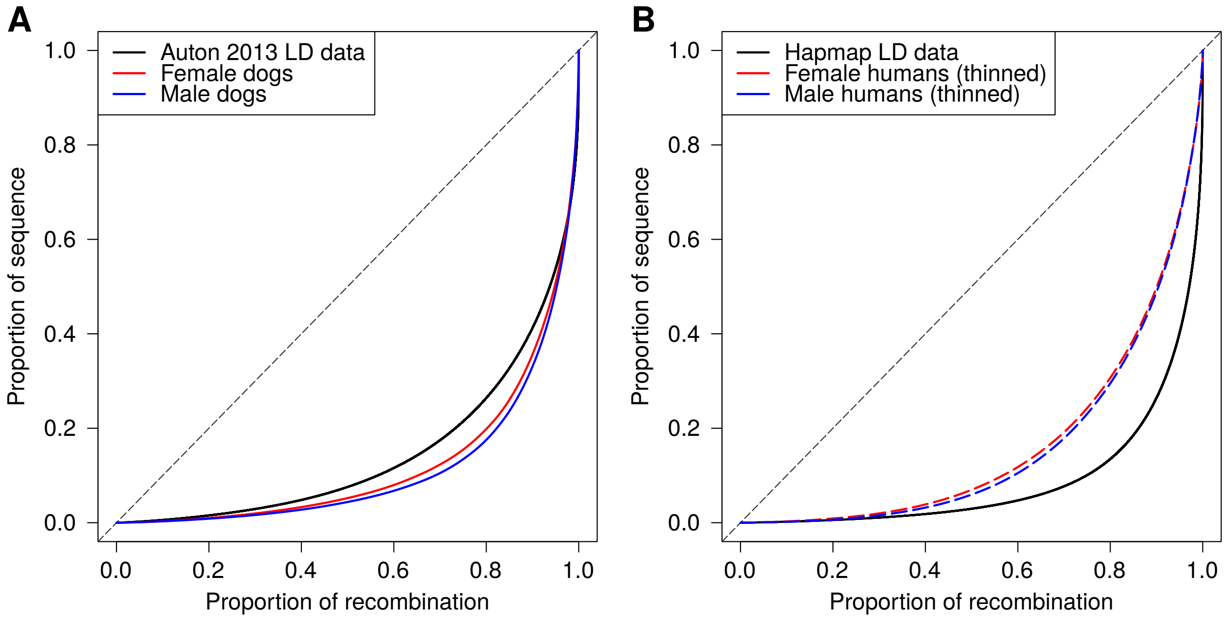

Figure S12: Estimates for the proportion of recombination occupying various proportions of the sequence differ between LD- and pedigree-based maps. For dogs (A), the LD data[1] (black line) appears to be more uniform than the pedigree-based estimates. In humans (B), the LD data[4] (black line) appears to be less uniform than the thinned pedigree-based estimates.
