# Supplementary material for: A Pedigree-Based Map of Recombination in the Domestic Dog Genome
Source: G3 (Bethesda). 2016 Sep 2;6(11):3517–24. doi: 10.1534/g3.116.034678 (PMC5100850; doi:10.1534/g3.116.034678)
Supplement: Supplemental Material [file supp_g3.116.034678_FigureS13.pdf]

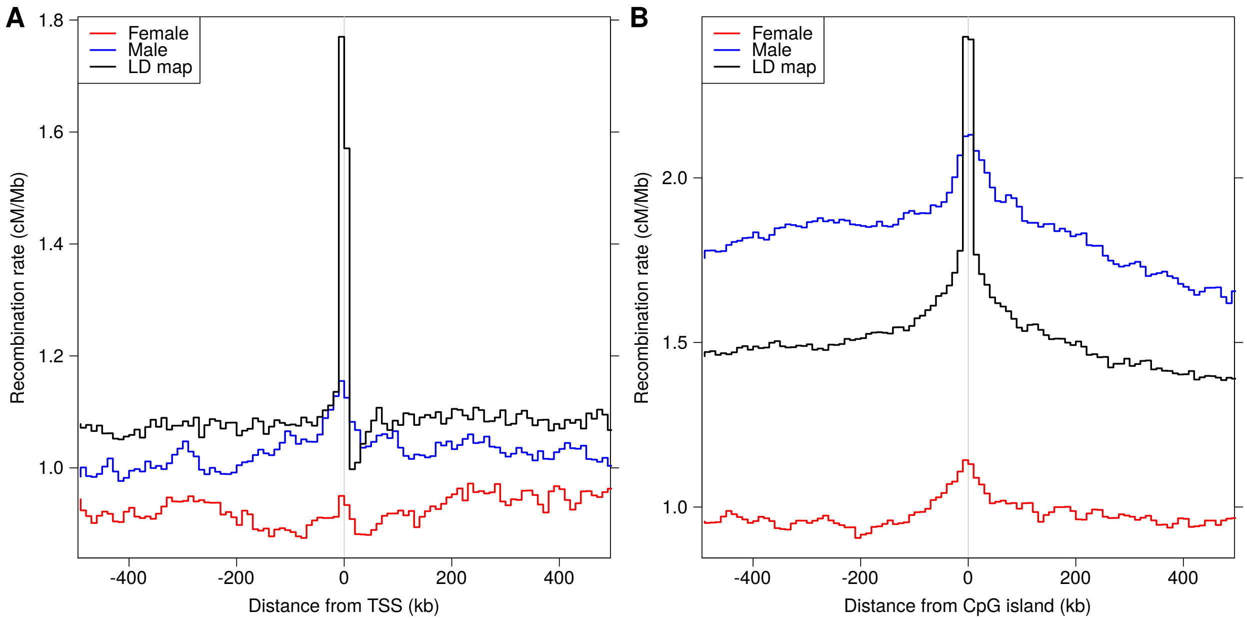

Figure S13: Sex differences in recombination around the TSS (A) and CpG islands (B). Female rates are shown red, male in blue. LD-based estimates[1] are shown in black. Recombination rates were estimated in 10 kb windows.
