# Supplementary material for: A Pedigree-Based Map of Recombination in the Domestic Dog Genome
Source: G3 (Bethesda). 2016 Sep 2;6(11):3517–24. doi: 10.1534/g3.116.034678 (PMC5100850; doi:10.1534/g3.116.034678)
Supplement: Supplemental Material [file supp_g3.116.034678_FigureS14.pdf]

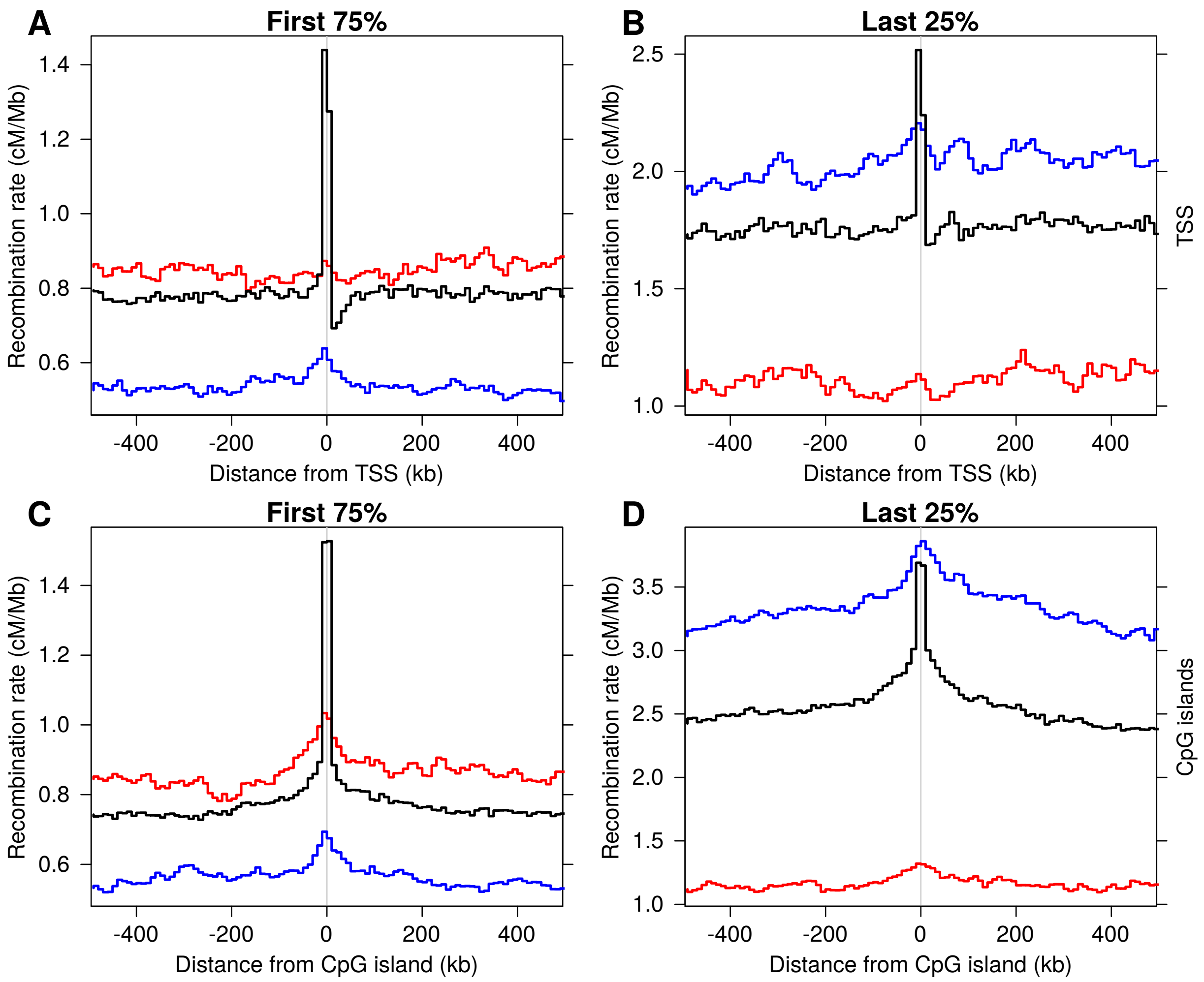

Figure S14: Recombination around TSS and CpG islands partitioned by chromosome position. Male rates are in blue, female in red, rates from the LD map in black. Rates were estimated in 10 kb bins. Rates were estimated for each feature by taking the centromeric 75% (A and C) and telomeric 25% (B and D) of each chromosome separately.
