# Supplementary material for: A Pedigree-Based Map of Recombination in the Domestic Dog Genome
Source: G3 (Bethesda). 2016 Sep 2;6(11):3517–24. doi: 10.1534/g3.116.034678 (PMC5100850; doi:10.1534/g3.116.034678)
Supplement: Supplemental Material [file supp_g3.116.034678_FigureS15.pdf]

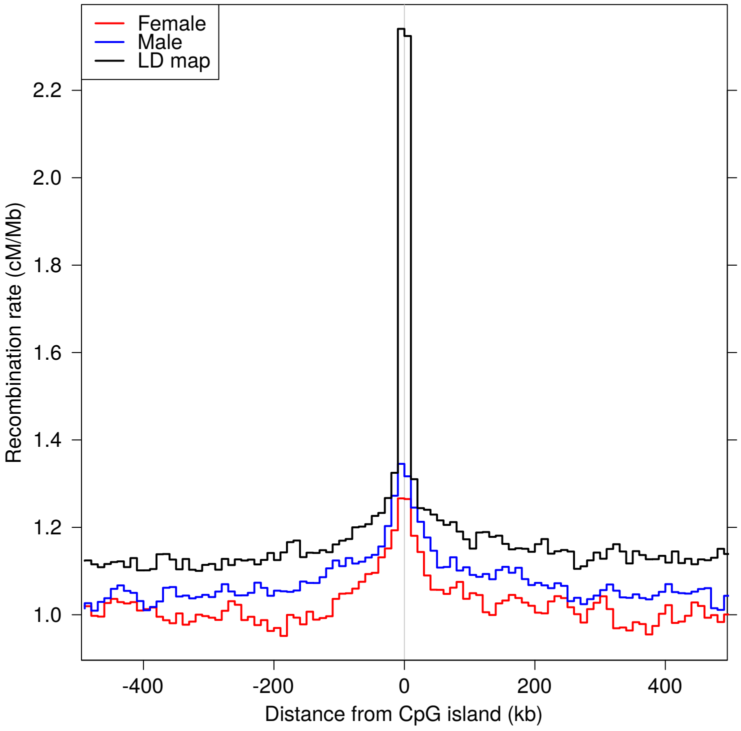

Figure S15: Recombination around a thinned subset of CpG islands. Male rates are in blue, female in red, rates from the LD map in black. Rates were estimated in 10 kb bins. CpG islands were thinned to a uniform distribution by keeping a maximum of 5 per non-overlapping 500 kb window.
