# Supplementary material for: A Pedigree-Based Map of Recombination in the Domestic Dog Genome
Source: G3 (Bethesda). 2016 Sep 2;6(11):3517–24. doi: 10.1534/g3.116.034678 (PMC5100850; doi:10.1534/g3.116.034678)
Supplement: Supplemental Material [file supp_g3.116.034678_FigureS16.pdf]

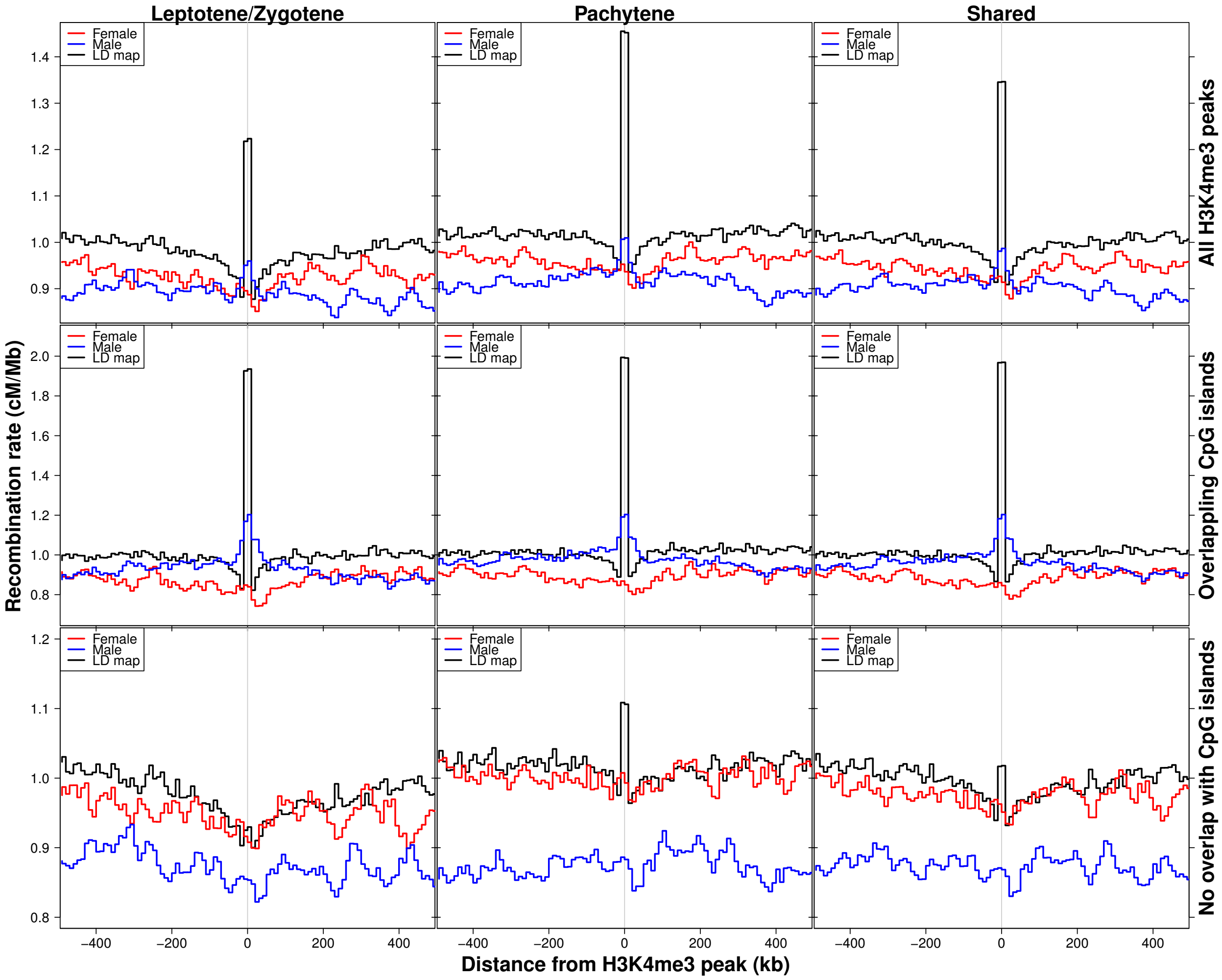

Figure S16: Recombination rate around H3K4 trimethylation marks found in dog spermatocytes of varying stages[1]. Male rates are shown in blue, female in red, and rates from the LD-based map in black. The left column of plots shows peaks in early stages of meiosis (leptotene/zygotene), the middle column pachytene, and the right column the union of all peaks. Similarly, the top row includes all peaks, while the middle and bottom rows show peaks with and without overlap with CpG islands, respectively. Rates were estimated in 10 kb bins for all plots.
