# Supplementary material for: A Pedigree-Based Map of Recombination in the Domestic Dog Genome
Source: G3 (Bethesda). 2016 Sep 2;6(11):3517–24. doi: 10.1534/g3.116.034678 (PMC5100850; doi:10.1534/g3.116.034678)
Supplement: Supplemental Material [file supp_g3.116.034678_TableS2.pdf]

CFA	Physical (bp)	First position (bp)	Last position (bp)	Female (cM)	Mean female rate (cM/Mb)	Male (cM)	Mean male rate (cM/Mb)	Sex avg. (cM)	Sex avg. rate (cM/Mb)	No. markers
1	122,678,785	4,283,592	122,309,715	95.54	0.78	85.17	0.69	90.12	0.73	8284
2	85,426,708	3,621,442	85,062,551	78.36	0.92	64.77	0.76	71.66	0.84	5550
3	91,889,043	5,604,604	91,556,345	75.63	0.82	62.64	0.68	68.77	0.75	6681
4	88,276,631	5,840,941	87,934,673	76.46	0.87	55.85	0.63	66.10	0.75	6231
5	88,915,250	1,243,143	88,673,195	95.67	1.08	68.00	0.76	81.70	0.92	6432
6	77,573,801	455,434	77,489,595	67.30	0.87	59.17	0.76	63.11	0.81	5320
7	80,974,532	180,153	80,809,723	70.31	0.87	47.59	0.59	58.81	0.73	5778
8	74,330,416	2,763,496	72,510,424	60.10	0.81	57.07	0.77	57.83	0.78	4846
9	61,074,082	876,259	60,812,630	62.66	1.03	50.65	0.83	55.67	0.91	4293
10	69,331,447	2,125,046	69,293,175	66.85	0.96	55.26	0.80	61.17	0.88	4573
11	74,389,097	4,087,888	74,253,347	58.71	0.79	48.36	0.65	53.60	0.72	4557
12	72,498,081	82,400	72,115,946	61.01	0.84	52.90	0.73	55.91	0.77	5364
13	63,241,923	4,067,434	62,932,928	54.05	0.85	45.71	0.72	49.52	0.78	4749
14	60,966,679	7,309,849	60,600,364	51.49	0.84	45.22	0.74	48.11	0.79	4153
15	64,190,966	4,913,124	64,007,939	48.69	0.76	44.57	0.69	46.43	0.72	4372
16	59,632,846	6,692,748	58,967,916	52.23	0.88	37.86	0.63	45.03	0.76	3829
17	64,289,059	5,285,642	63,501,532	63.44	0.99	50.07	0.78	56.58	0.88	4731
18	55,844,845	3,203,856	55,355,125	55.33	0.99	52.01	0.93	53.71	0.96	4014
19	53,741,614	3,189,264	53,349,320	53.22	0.99	49.99	0.93	52.29	0.97	3733
20	58,134,056	4,356,904	58,000,062	52.64	0.91	55.38	0.95	53.59	0.92	4140
21	50,858,623	4,450,666	50,719,350	51.66	1.02	44.16	0.87	47.84	0.94	3706
22	61,439,934	2,513,263	61,217,407	52.21	0.85	49.67	0.81	50.57	0.82	4422
23	52,294,480	1,203,392	52,291,577	49.31	0.94	44.47	0.85	46.63	0.89	3928
24	47,698,779	1,029,209	47,233,919	49.71	1.04	53.74	1.13	51.50	1.08	3625
25	51,628,933	6,038,820	51,469,123	56.49	1.09	48.57	0.94	52.44	1.02	3922
26	38,964,690	2,407,850	38,657,286	46.66	1.20	41.16	1.06	43.81	1.12	2853
27	45,876,710	444,525	42,191,669	48.31	1.05	48.70	1.06	47.01	1.02	3354
28	41,182,112	4,526,049	40,963,512	51.11	1.24	39.62	0.96	45.41	1.10	3074
29	41,845,238	913,671	41,543,120	49.96	1.19	42.06	1.01	44.75	1.07	3017
30	40,214,260	5,433,983	39,826,282	43.89	1.09	37.30	0.93	40.20	1.00	2942
31	39,895,921	580,882	39,466,279	51.82	1.30	38.07	0.95	44.32	1.11	2855
32	38,810,281	114,049	37,999,255	46.13	1.19	41.68	1.07	43.78	1.13	2746
33	31,377,067	447,033	30,994,965	45.46	1.45	36.01	1.15	40.75	1.30	2320
34	42,124,431	624,977	41,979,553	48.69	1.16	38.71	0.92	43.36	1.03	3239
35	26,524,999	1,164,664	26,257,078	41.87	1.58	30.59	1.15	35.88	1.35	2171
36	30,810,995	251,708	30,523,428	41.77	1.36	29.61	0.96	35.95	1.17	2286
37	30,902,991	1,364,851	30,583,437	45.59	1.48	36.18	1.17	40.42	1.31	2333
38	23,914,537	246,006	23,695,770	41.67	1.74	27.38	1.15	33.68	1.41	1895
			**Total**	2161.99	0.98	1815.91	0.82	1978.01	0.90	156,318

Table S2: Physical and genetic chromosome lengths.
