# Supplementary material for: A Pedigree-Based Map of Recombination in the Domestic Dog Genome
Source: G3 (Bethesda). 2016 Sep 2;6(11):3517–24. doi: 10.1534/g3.116.034678 (PMC5100850; doi:10.1534/g3.116.034678)
Supplement: Supplemental Material [file supp_g3.116.034678_FigureS17.pdf]

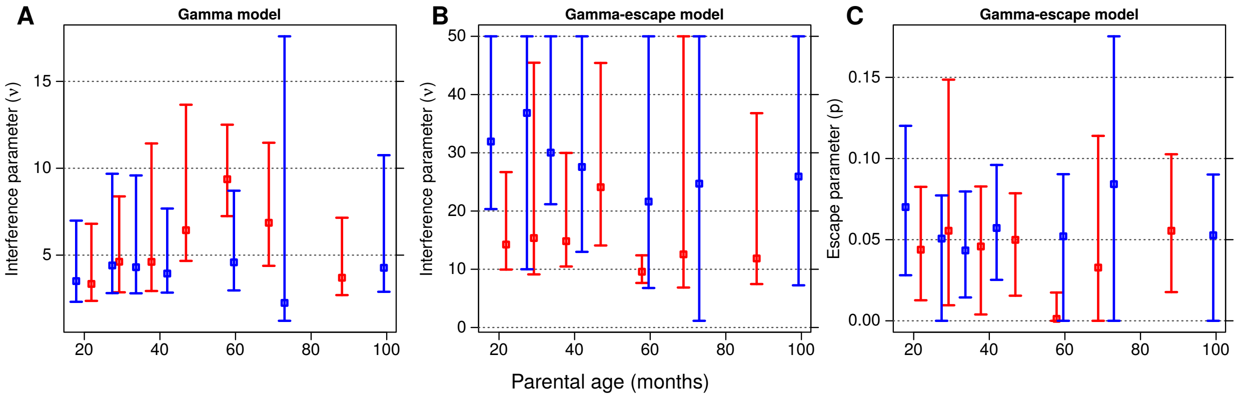

Figure S17: Estimates of crossover interference parameters in the dog genome as a function of age. Dog meioses were partitioned into 7 approximately equal sized bins on the basis of parental age at birth. Interference strength for the simple gamma model is shown in A. The parameters for the Housworth-Stahl gamma-escape model are shown in B (interference strength) and C (escape). Males are shown in blue and females in red. The error bars represent a 95% confidence interval estimated from 100 bootstrap iterations.
