# Supplementary material for: A Pedigree-Based Map of Recombination in the Domestic Dog Genome
Source: G3 (Bethesda). 2016 Sep 2;6(11):3517–24. doi: 10.1534/g3.116.034678 (PMC5100850; doi:10.1534/g3.116.034678)
Supplement: Supplemental Material [file supp_g3.116.034678_FigureS18.pdf]

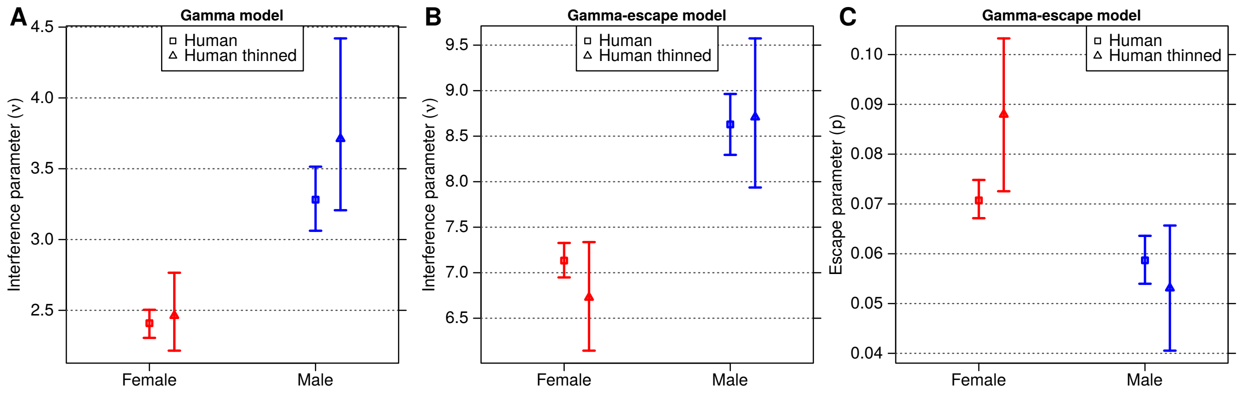

Figure S18: Estimates of crossover interference parameters in the human genome. Here, the human data is thinned according to the procedure outlined in the Methods section, reducing the meiosis count and number of SNPs to more closely resemble what is found in dogs. Interference strength for the simple gamma model is shown in A. The parameters for the Housworth-Stahl gamma-escape model are shown in B (interference strength) and C (escape). Males are shown in blue and females in red. Estimates for the full resolution human data are shown in boxes, and the thinned human data in triangles. The error bars represent a 95% confidence interval estimated from 1000 bootstrap iterations.
