# Supplementary material for: A Pedigree-Based Map of Recombination in the Domestic Dog Genome
Source: G3 (Bethesda). 2016 Sep 2;6(11):3517–24. doi: 10.1534/g3.116.034678 (PMC5100850; doi:10.1534/g3.116.034678)
Supplement: Supplemental Material [file supp_g3.116.034678_TableS3.pdf]

	Size (kb)	Male rate	Female rate	Difference (male - female)
TSS upstream	400	1.01	0.92	0.09
TSS	50	1.12	0.92	0.20
TSS downstream	400	1.03	0.94	0.09
CpG upstream	400	1.84	0.95	0.89
CpG island	50	2.09	1.11	0.98
CpG downstream	400	1.75	0.97	0.78

Table S3: Recombination rate around TSS and CpG islands. Recombination rates are given in cM/Mb and were estimated in 10kb bins, and averaged over the indicated window. Rates surrounding CpG islands represent a 50kb window centered on the CpG island, while rates for the TSS are given as a 50kb bin ending at the TSS, capturing the elevated rate immediately upstream.
