# Supplementary material for: A Pedigree-Based Map of Recombination in the Domestic Dog Genome
Source: G3 (Bethesda). 2016 Sep 2;6(11):3517–24. doi: 10.1534/g3.116.034678 (PMC5100850; doi:10.1534/g3.116.034678)
Supplement: Supplemental Material [file supp_g3.116.034678_FigureS1.pdf]

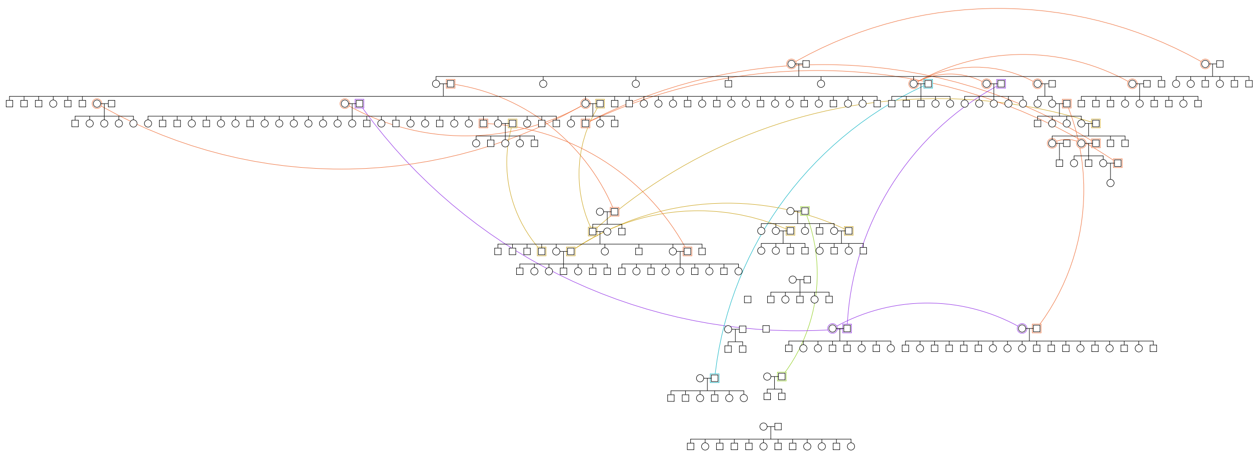

Figure S1: Structure of the dog pedigree. Males are represented by squares, females by circles. Colored lines indicate individuals repeated on the plot, that are involved in more than one mating pair.
