# Supplementary figures and images for: A Pedigree-Based Map of Recombination in the Domestic Dog Genome
Source: G3 (Bethesda). 2016 Sep 2;6(11):3517–24. doi: 10.1534/g3.116.034678 (PMC5100850; doi:10.1534/g3.116.034678)

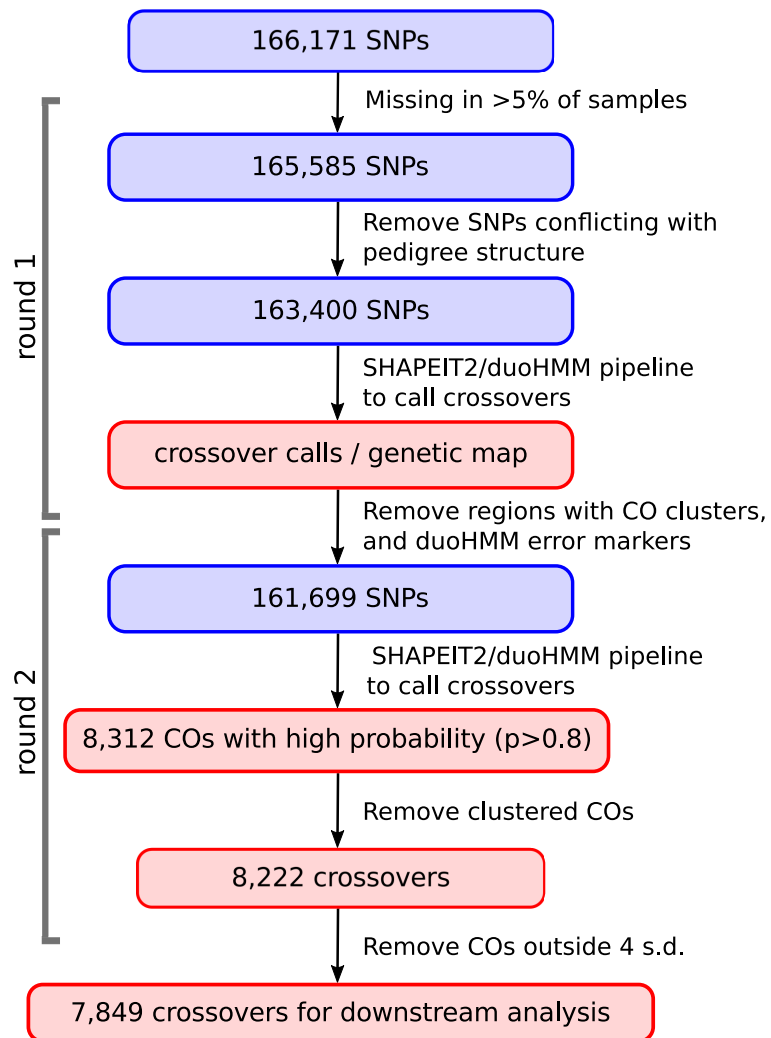

Figure S2: Overview of the analysis pipeline. CO, crossovers; s.d., standard deviation.

Supplement: Supplemental Material [file supp_g3.116.034678_FigureS2.pdf]
