# Supplementary material for: A Pedigree-Based Map of Recombination in the Domestic Dog Genome
Source: G3 (Bethesda). 2016 Sep 2;6(11):3517–24. doi: 10.1534/g3.116.034678 (PMC5100850; doi:10.1534/g3.116.034678)
Supplement: Supplemental Material [file supp_g3.116.034678_FigureS3.pdf]

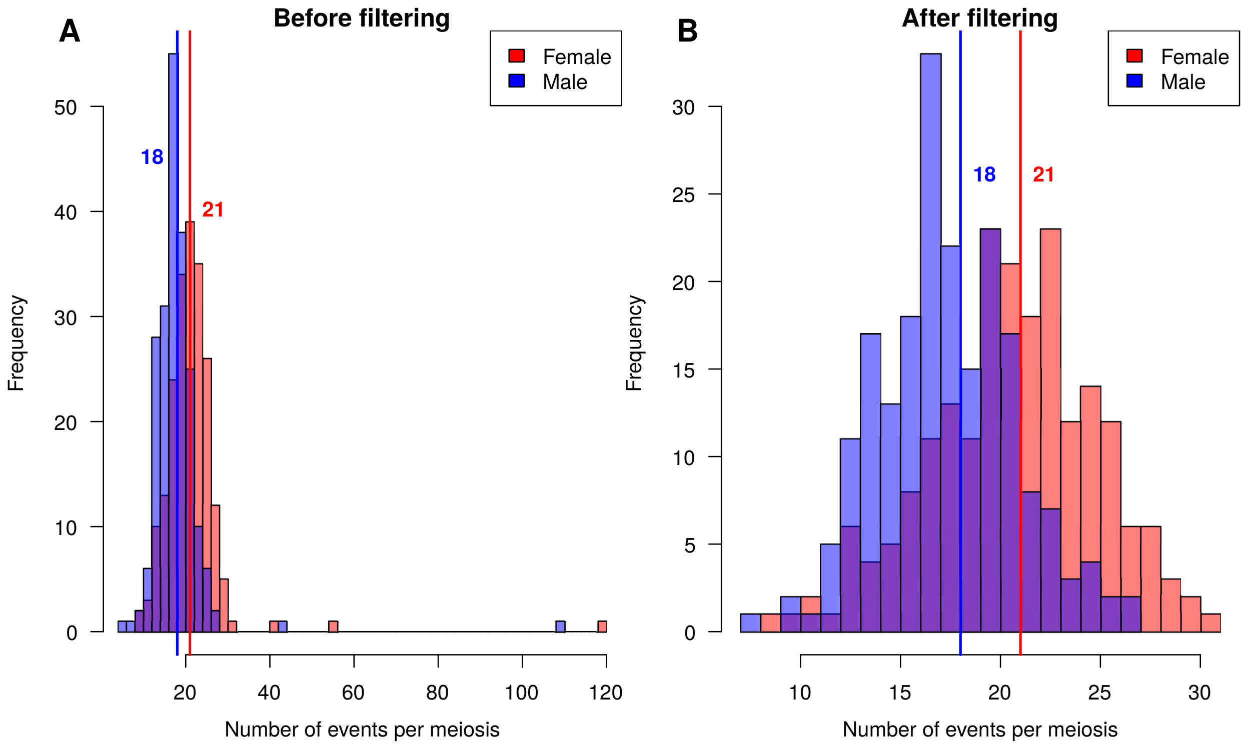


Figure S3: Distribution of the number of crossover events per meiosis. Panel A shows all meioses prior to filtering (n=414), and panel B shows the filtered set (n=408). Female meioses are shown in red, males in blue.
