# Supplementary material for: A Pedigree-Based Map of Recombination in the Domestic Dog Genome
Source: G3 (Bethesda). 2016 Sep 2;6(11):3517–24. doi: 10.1534/g3.116.034678 (PMC5100850; doi:10.1534/g3.116.034678)
Supplement: Supplemental Material [file supp_g3.116.034678_FigureS4.pdf]

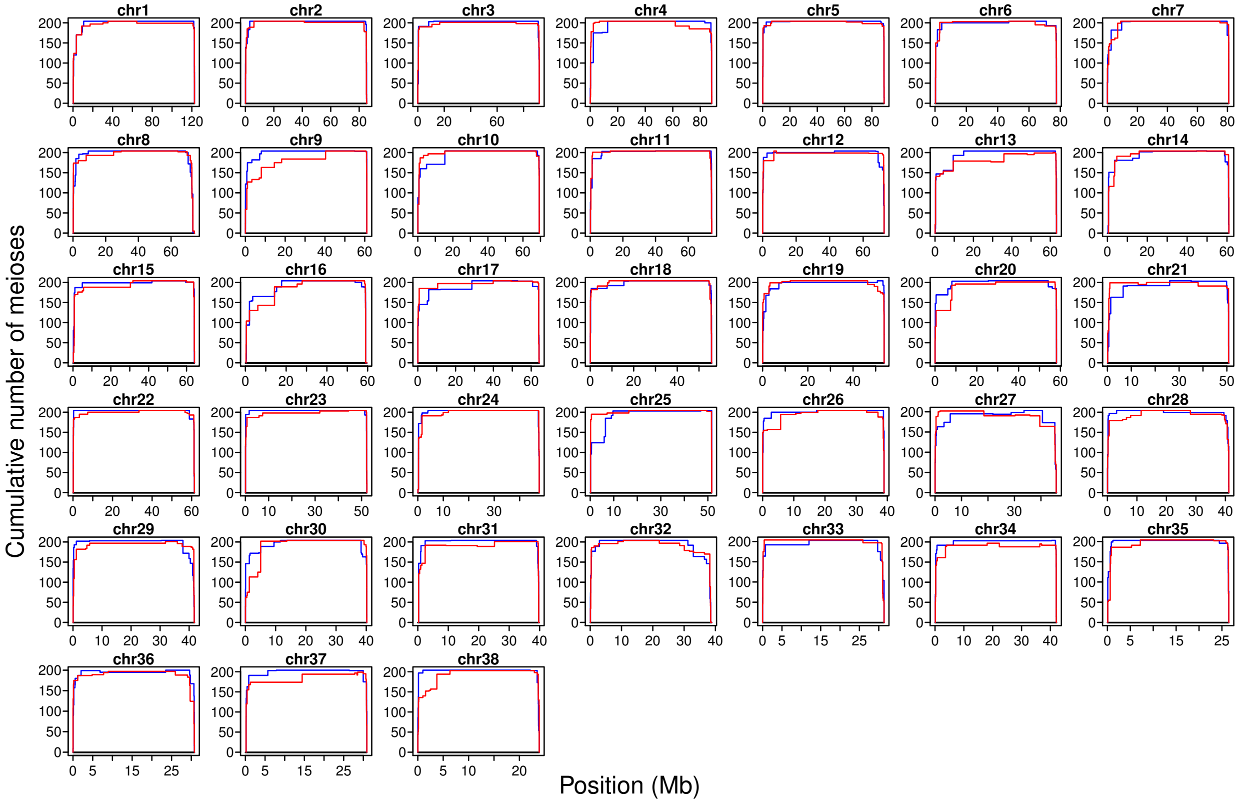

Figure S4: The effective number of meioses as a function of physical position is shown along each chromosome. Red curves represent females (n=204), blue curves represent males (n=204).
