# Supplementary material for: A Pedigree-Based Map of Recombination in the Domestic Dog Genome
Source: G3 (Bethesda). 2016 Sep 2;6(11):3517–24. doi: 10.1534/g3.116.034678 (PMC5100850; doi:10.1534/g3.116.034678)
Supplement: Supplemental Material [file supp_g3.116.034678_FigureS5.pdf]

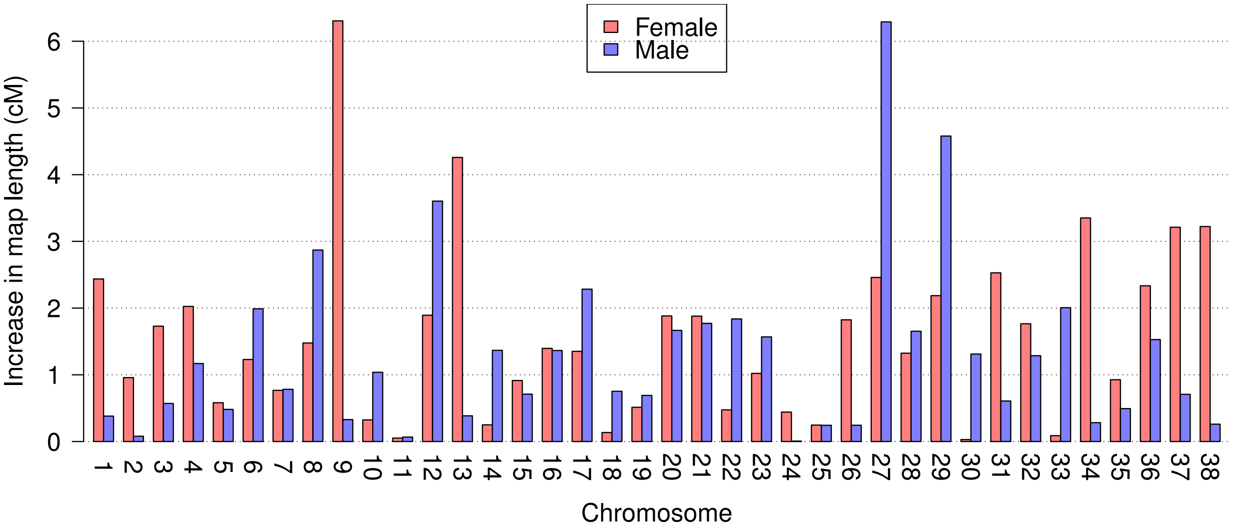

Figure S5: Increase in map length in each chromosome after accounting for the effective number of meioses. Each bar represents the difference in map length after taking into account a reduced number of observable meioses towards chromosome ends compared to the map length calculated using a fixed number of meioses (n=204 for females in red, n=204 for males, blue).
