# Supplementary material for: A Pedigree-Based Map of Recombination in the Domestic Dog Genome
Source: G3 (Bethesda). 2016 Sep 2;6(11):3517–24. doi: 10.1534/g3.116.034678 (PMC5100850; doi:10.1534/g3.116.034678)
Supplement: Supplemental Material [file supp_g3.116.034678_FigureS6.pdf]

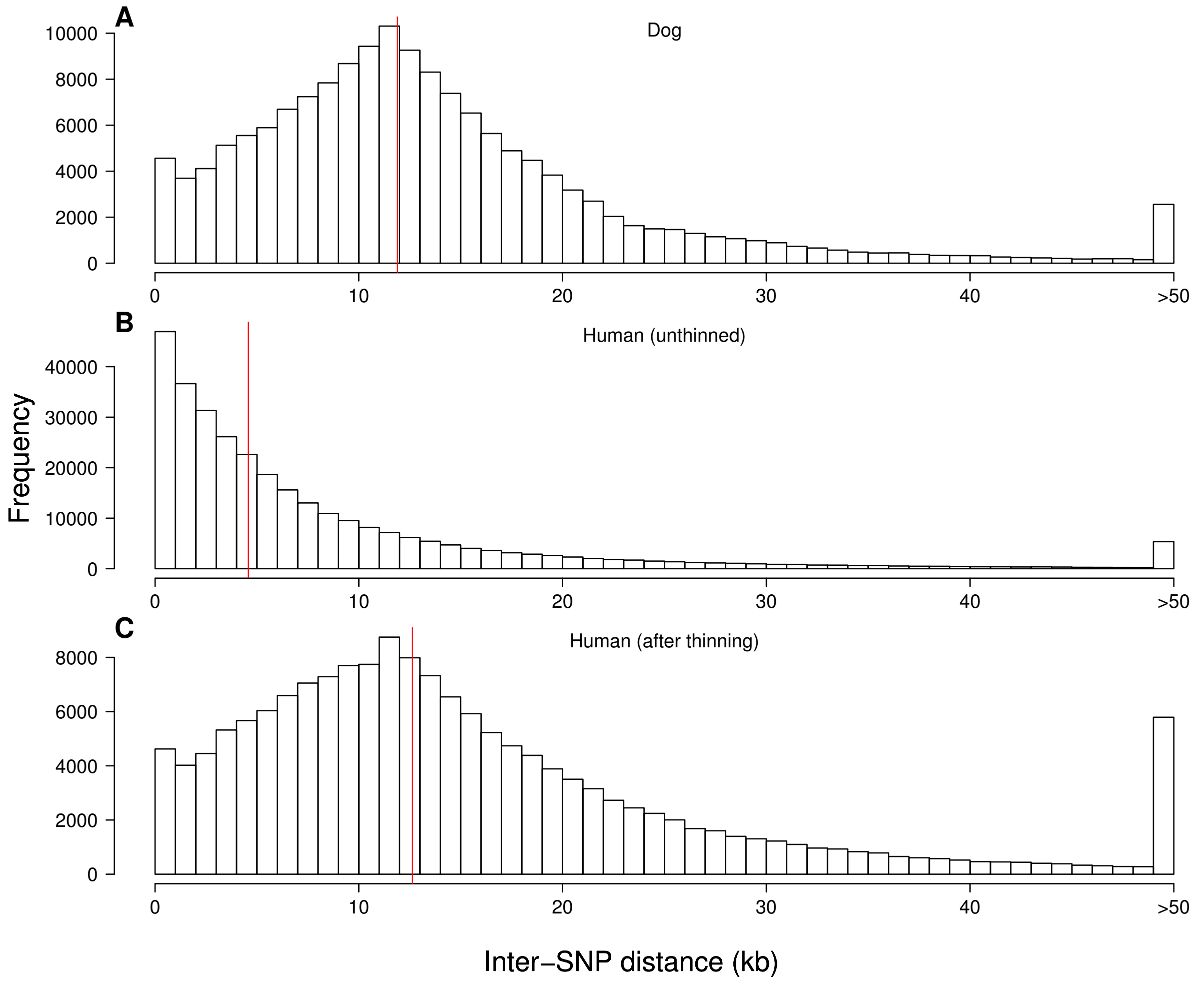

Figure S6: Distribution of inter-SNP distances in the dog data (A), the human data prior to thinning (B), and the human data after the thinning procedure (C). The red line represents the median inter-SNP distance for each distribution.
